# Neurosteroids as a prospective treatment for social anxiety disorder

**DOI:** 10.1017/S1092852925100485

**Published:** 2025-08-11

**Authors:** Richard Skaff

**Affiliations:** Independent Scholar, Palos Verdes Peninsula, CA, USA

Anxiety is one of the most prevalent mental health difficulties in the United States and worldwide, afflicting millions of people in varying degrees, whether it is generalized anxiety disorder, social anxiety disorder, or any other anxiety-related disorder.

In 2014, *CNS Spectrums* published an article titled **“Neurobiological Correlates of Social Anxiety Disorder: An Update.”** In this publication, the authors offered various explanations for the underlying mechanisms of social anxiety disorder (SAD), emphasizing the central role of the amygdala. They proposed that the amygdala is critically involved in fear conditioning and emotional processing, and noted that individuals with SAD tend to exhibit exaggerated amygdala responses to social–emotional stimuli.^1^

Beyond the amygdala, the article also highlighted the involvement of other brain regions implicated in SAD-related neural circuitry. These include the medial dorsal raphe, striatum, locus coeruleus, prefrontal cortex, insular cortex, and anterior cingulate cortex. The complexity and interconnectedness of these areas complicate targeted treatment strategies, as each plays a different role in emotional regulation and cognitive processing.^1^

Meanwhile, pharmaceutical companies continue their pursuit of novel treatments for psychiatric conditions such as anxiety, depression, and psychosis. Many of these strategies have historically relied on manipulating levels of well-known neurotransmitters (e.g., serotonin, dopamine, norepinephrine). However, a major limitation of this approach lies in the absence of a standardized baseline for what constitutes “normal” levels of these neurotransmitters. Furthermore, there is currently no clinical test capable of precisely measuring a deficiency or excess of neurotransmitters, nor is it possible to directly assess how such imbalances affect specific brain circuits or gene expression.

Interestingly, the pharmaceutical industry has also revisited certain substances previously explored during controversial experiments, such as the CIA’s MK-Ultra program—namely, hallucinogens and dissociative agents. Modified versions of these compounds are now being used, and others are re-evaluated for therapeutic potential in treatment-resistant cases of depression and anxiety. These drugs, under physician supervision, may offer short-term relief by inducing mild dissociative or altered states of consciousness, effectively disrupting the person’s ongoing emotional distress. Long-term results and ramifications of these treatments remain undisclosed or unknown.

More recently, the concept of **neurosteroids** has regained attention as a potential therapeutic avenue, particularly for targeting the amygdala and modulating emotions such as fear and anxiety.

Neurosteroids are a class of steroids synthesized within the brain, capable of rapidly modulating neuronal excitability via nongenomic mechanisms. Neurosteroids are derived from circulating steroid hormones and are primarily synthesized in the hippocampus and other brain regions.^2^ They can be classified into different structural categories, such as:

- **Pregnane neurosteroids**: e.g., allopregnanolone and THDOC
- **Androstane neurosteroids**: e.g., androstanediol (with anxiolytic and anticonvulsant properties)
- **Sulfated neurosteroids**: e.g., pregnenolone sulfate (PS) and dehydroepiandrosterone sulfate (DHEAS)^3^

One notable development in the neurosteroid and intranasal treatment space is **Vistagen’s “Fasedienol,”** an investigational pherine nasal spray currently in Phase 3 clinical development in the U.S. for the acute treatment of SAD in adults. Unlike traditional antianxiety medications, Fasedienol operates through a novel mechanism of action. When administered intranasally in microgram doses, it activates receptors in the nasal passages, which stimulate specific neurons in the olfactory bulbs. These, in turn, modulate neural pathways connected to the limbic amygdala—one of the key regions involved in SAD.^4^

Crucially, fasedienol produces anxiolytic effects without necessitating systemic distribution or direct activity on central brain neurons. This allows for a rapid-onset and short-duration therapeutic effect with a potentially improved safety profile. In late 2023, Vistagen announced positive results from its PALISADE-2 Phase 3 trial, where fasedienol met all primary, secondary, and exploratory endpoints. In 2024, the company-initiated PALISADE-3 and plans to launch PALISADE-4, along with additional registration-enabling further studies.^4^

The likely benefits of targeting neurosteroids through innovative delivery method would be the following:

- Natural regulation of anxiety without strong sedation or dependency.
- Quicker onset of effects compared to traditional antidepressants, which could take 4–6 weeks to obtain therapeutic results.
- Novel alternative for patients who do not respond to SSRIs or benzodiazepines.

While treatment of refractory depression and anxiety has been a daunting task for prescribers, the quest for the perfect elixir to cure various psychiatric disorders is ongoing. In a psychiatric field of uncertainty and speculation, novel approaches to treatment are always welcome. Hopefully, neurosteroids might denote an encouraging edge in understanding and treating anxiety, if it is truly based on science rather than marketing and on clinically significant evidence that will indicate better outcomes with minimal harm to the patient.
